# Discontinuity Preserving Image Registration through Motion Segmentation: A Primal-Dual Approach

**DOI:** 10.1155/2016/9504949

**Published:** 2016-09-19

**Authors:** Silja Kiriyanthan, Ketut Fundana, Tahir Majeed, Philippe C. Cattin

**Affiliations:** Medical Image Analysis Center, Department of Biomedical Engineering, University of Basel, Basel, Switzerland

## Abstract

Image registration is a powerful tool in medical image analysis and facilitates the clinical routine in several aspects. There are many well established elastic registration methods, but none of them can so far preserve discontinuities in the displacement field. These discontinuities appear in particular at organ boundaries during the breathing induced organ motion. In this paper, we exploit the fact that motion segmentation could play a guiding role during discontinuity preserving registration. The motion segmentation is embedded in a continuous cut framework guaranteeing convexity for motion segmentation. Furthermore we show that a primal-dual method can be used to estimate a solution to this challenging variational problem. Experimental results are presented for MR images with apparent breathing induced sliding motion of the liver along the abdominal wall.

## 1. Introduction

Image registration became an indispensable tool for many medical applications including the Image-Guided Therapy systems. Today's image registration methods [1] are powerful at handling rigid as well as nonrigid motion on mono- and multimodal images. With the introduction of imaging technologies capable of capturing 4D organ motion, such as 4D-MRI [2], 4D-CT, or 4D-Ultrasound, imagery of sliding organs became a significant focus of research. Most of the state-of-the-art image registration methods cannot yet properly deal with these data sets, as their regularization causes continuous motion fields over the organ boundaries.

In recent publications, the concept of the direction-dependent regularization of the displacement field has been introduced; see, for example, [3]. The drawback of these methods is the necessity of providing a good manual segmentation of the boundaries. Approaches that do not rely on prior manual segmentations have been a topic of research for some decades in classical computer vision such as optical flow. Transfer of these research results into the medical field was however scarce. Already in 1989, Mumford and Shah [4] proposed in their pioneering work a functional for image segmentation that avoids spatial smoothing in certain locations of the image, thus preserving discontinuities. Some years later, Weickert and Schnörr [5] proposed an extension of nonquadratic variational regularization for discontinuities preserving optical flow that uses spatiotemporal regularizers instead of flow-driven spatial smoothness. The resulting convex function guaranteed global convergence that could be solved with standard gradient based optimisation schemes. Vese and Chan [6] then introduced a level set framework based approach to efficiently solve the Mumford and Shah minimization problem for segmentation. Another influential approach based on the Total Variation (TV) norm, known to preserve discontinuities, was proposed by Rudin et al. [7], the ROF model. The beneficial behaviour of the TV norm was also exploited in recent registration and optical flow methods, as, for example, by [8, 9].

Motion segmentation has been a topic of research for quite some time. In 1993, Nesi [10] proposed a variational optical flow approach that incorporates a variable for the presence of discontinuities and leads to a piecewise smooth motion estimation. Cremers and Schnörr introduced in [11] a variational approach seamlessly integrating motion segmentation and shape regularization into one single energy functional. In [12], the authors formulated the motion segmentation problem in a Bayesian inference framework, whereas the motion discontinuity could be represented either as an explicit spline function or by an implicit level set formulation allowing for an arbitrary number of objects to segment. As motion discontinuities mainly appear at object boundaries, it seems natural to combine motion segmentation and registration. Given a good motion segmentation, a proper discontinuous motion field can be estimated and vice versa. Amiaz and Kiryati [13] tried to leverage this property by combining motion segmentation with the optical flow method of Brox et al. [8] in a level set framework. They showed that motion segmentation leads to better discontinuities in the motion field. However, their approach highly depends on the initialisation for the motion segmentation and displacement field. Furthermore, the level set approach is prone to local minima and a rather good initialisation has to be provided in advance.

In this paper we propose an elastic registration approach that handles discontinuities in the motion field by combining motion segmentation and registration in a variational scheme. For motion segmentation we make use of the so-called “continuous cuts” [14, 15] scheme that guarantees a globally optimal solution for a fixed motion field. In contrast to our previous work [16], the proposed energy functional is this time TV-*L* ^1^ regularized for both the motion segmentation and the displacement field and the fidelity term is formulated as a sum of absolute values of the constraints. Minimizing the TV-*L* ^1^-regularized functionals is, however, inherently difficult due to the nonsmoothness of the TV. This is an active field of research, such as [17–20] and the references therein which are mainly based on operator splitting methods in convex analysis that split energy functionals into the nonlinear and linear terms. We show how to solve the proposed complex energy functional with the primal-dual method of Chambolle and Pock [17], which is very efficient for a wide class of nonsmooth problems. The proposed method is then applied to register 2D MR image pairs with apparent breathing induced sliding motion of the liver along the abdominal wall. The preliminary version of this paper has been published in [21].

## 2. Method

In this section we describe the proposed registration method which integrates the displacement field estimation into the convex segmentation method of Chan et al. [14], in order to find smooth displacement fields whilst preserving the discontinuities.

### 2.1. Registration

Let *Ω* ⊂ *ℝ* ^2^ be the domain of the pixel positions **x** = (*x*_1_, *x*_2_). We then define by the functions *R* : *Ω* → *ℝ* and *T* : *Ω* → *ℝ* our reference and template image. The aim of image registration is to find a transformation Φ(**x**)≔**x** + **w**(**x**) such that the relation *T*∘Φ ≈ *R* holds. The function

(1) $$\begin{array}{c} w:\left\{\begin{array}{cc} \Omega ⟶R^{2} & \\ x⟼w\left(x\right)≔\left(u\left(x\right),v\left(x\right)\right), & \end{array}\right. \end{array}$$

with *u*, *v* : *Ω* → *ℝ*, describes the displacement field and will be the intrinsic function we investigate. For convenience we will use the abbreviations **w**, *u*, and *v* for **w**(**x**), *u*(**x**), and *v*(**x**).

To solve a nonrigid registration problem with expected discontinuities in the displacement field, we want to follow a variational approach and we therefore first consider the energy functional

(2) $$\begin{array}{c} E_{fs}\left(\;w\;\right)=\int_{\Omega }f\left(\;w\;\right)+\mu s\left(\;w\;\right)dx, \end{array}$$

where *μ* ∈ *ℝ* ^+^ is a weighting parameter and *f* and *s* are the fidelity term and the smoothness term, respectively, which are defined as

(3) fwfu,v≔γ1Tx+w−Rx+γ2∂x1Tx+w−∂x1Rx+γ2∂x2Tx+w−∂x2Rx,

(4) swsu,v≔∇u2+∇v2=∇w.

The fidelity term *f* incorporates the constraints for the grey value constancy and the gradient constancy and the corresponding weighting parameters are given by *γ*_1_, *γ*_2_ ∈ *ℝ*_0_^+^. The smoothness term *s* results in the *L* ^1^-norm and, respectively, the vectorial TV of **w**.

The energy functional *E*_*fs*_ is motivated by the energy functional proposed by Brox et al. [8]. Instead of using the approximation function Ψ for the *L* ^1^ norm, here the pure *L* ^1^ norm is used for the smoothness term *s* and for the fidelity term *f* the sum of the absolute grey value difference and the componentwise absolute gradient differences is taken.

### 2.2. Motion Segmentation

Now we would like to integrate the formulation of the energy functional above into the convex segmentation model of Chan et al. [14]. To differentiate the displacement field **w** into **w** ^+^ and **w** ^−^, we therefore choose a binary function

(5) $$\begin{array}{c} \tilde{u}:\left\{\begin{array}{cc} R^{2}⟶\left\{0,1\right\} & \\ x⟼\tilde{u}\left(x\right)≔1_{\Sigma }\left(x\right), & \end{array}\right. \end{array}$$

where Σ⊆*Ω*⊆*ℝ* ^2^, with $\Sigma ≔\left\{x\in \Omega |\tilde{u}\left(x\right)=1\right\}$ . By defining *D*(**w**)≔*f*(**w**) + *μs*(**w**) as a data term, we formulate our energy functional as

(6) Ew+,w−,u~=∫ΩDw+u~xdx+∫ΩDw−1−u~xdx+ν∫Ω∇u~xdx,

where the last term of the above energy is regularization defined by the TV norm and *ν* is a constant parameter.

The energy functional *E* (6) is very much related to the energy functional proposed by Amiaz and Kiryati [13]. Instead of applying the Heaviside function to a level set function, the binary function $\tilde{u}$ is used here. Furthermore the approximation function Ψ for the *L* ^1^ norm, which was used in [13], is omitted here. Instead the pure *L* ^1^ norm is used for the smoothness terms *s*(**w** ^±^) (4) and the fidelity terms *f*(**w** ^±^) are replaced by a sum of absolute values of the constraints (3).

As pointed out by Chan et al. in [14], (6) is strongly related to the Mumford-Shah functional [4] and can be written as

(7) E~w+,w−,Σ=∫ΣDw+dx+∫Ω∖ΣDw−dx+νPer⁡Σ;Ω,

where Per⁡(Σ; *Ω*) denotes the perimeter of the set Σ⊆*Ω*.

**Remark 1 .**

One can show that a global minimizer Σ_min_ of $\tilde{E}(w^{+},w^{-},\cdot )$ can be found by solving the convexified problem ${min}_{0\leq \tilde{u}\leq 1}E(w^{+},w^{-},\tilde{u})$ and finally setting $\Sigma =\Sigma (\eta )≔\left\{x\in \Omega |\tilde{u}(x)\geq \eta \right\}$ for almost every *η* with *η* ∈ [0,1] (see Proposition  1 in [16]). In fact, this holds for any data terms *D*(**w** ^+^) and *D*(**w** ^−^) that are measurable.

Finally, we obtain the aimed displacement field by setting $w≔w^{+}\tilde{u}+w^{-}(1-\tilde{u})$ .

**Figure 1:**
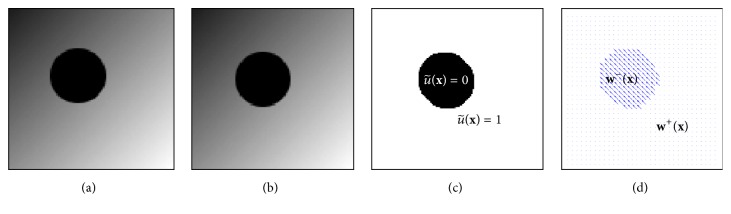
From (a) to (d): the reference and template image, the motion segmentation function $\tilde{u}$ , and the displacement field **w**.

In Figure 1 we illustrate our method by an example. The motion segmentation function $\tilde{u}$ splits the displacement field **w** into the two displacement fields **w** ^+^ and **w** ^−^.

## 3. Optimisation

### 3.1. Iterative Scheme

To facilitate the optimisation procedure we replace the fidelity term *f* in (3) by its linearised version

(8) $$\begin{array}{c} f\left(\;w\;\right)=\gamma_{1}\left|\;\rho_{1}\left(\;w\;\right)\;\right|+\gamma_{2}\left|\;\rho_{2}^{\left(1\right)}\left(\;w\;\right)\;\right|+\gamma_{2}\left|\;\rho_{2}^{\left(2\right)}\left(\;w\;\right)\;\right|, \end{array}$$

where

(9) ρ1wρ1x,w0,w≔Tx+w0+∇Tx+w0Tw−w0−Rx,ρ21wρ21x,w0,w≔∂x1Tx+w0+∂x1x1Tx+w0∂x2x1Tx+w0Tw−w0−∂x1Rx,ρ22wρ22x,w0,w≔∂x2Tx+w0+∂x1x2Tx+w0∂x2x2Tx+w0Tw−w0−∂x2Rx,

with **w**_0_ fixed.

The minimization of the energy functional *E* (6) with respect to **w** ^+^, **w** ^−^, and $\tilde{u}$ is then performed by the following iterative scheme:

(1) For fixed **w** ^+^ and **w** ^−^, solve

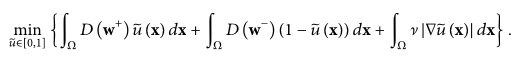

(10)

(2) For fixed $\tilde{u}$ , solve
(11) $$\begin{array}{c} \underset{w^{+}}{\min}\left\{\;\int_{\Omega }D\left(\;w^{+}\;\right)\tilde{u}\left(\;x\;\right)dx\;\right\}. \end{array}$$
(3) For fixed $\tilde{u}$ , solve
(12) $$\begin{array}{c} \underset{w^{-}}{\min}\left\{\;\int_{\Omega }D\left(\;w^{-}\;\right)\left(\;1-\tilde{u}\left(\;x\;\right)\;\right)dx\;\right\}. \end{array}$$

Although problem (10) is convex, it is important to note that the overall minimization of the energy functional *E* (6) is a nonconvex optimisation problem. For the minimization of nonconvex energy functionals there exist convex relaxation methods, which are able to provide solutions close to a global minimum. Recently, Strekalovskiy et al. [22] proposed such a method for nonconvex vector-valued labelling problems. In this paper however we will not make use of these kinds of methods.

Note that, compared to our previous work [16], this time we did not introduce an auxiliary $\tilde{v}$ in the energy functional. Therefore our iterative scheme consists of only 3 steps instead of 4. The reason for this change is that we intend to use a different numerical approach to solve our problem. More precisely, to solve subproblems (10), (11), and (12) in a fast and efficient way, we follow a primal-dual approach as described by Chambolle and Pock in [17]. We therefore recapitulate in the next section the basic notations and formulations.

### 3.2. Basic Framework for the Primal-Dual Approach of Chambolle and Pock

First, we define by *X* and *Y* two finite-dimensional real vector spaces. Their inner products are denoted by 〈·,·〉_*X*_ and 〈·,·〉_*Y*_, respectively, and their induced norms are given by ${\left\|\cdot \right\|}_{X}=\sqrt{{\langle \cdot ,\cdot \rangle }_{X}}$ and ${\left\|\cdot \right\|}_{Y}=\sqrt{\langle \cdot ,\cdot \rangle_{Y}}$ , respectively. The general nonlinear primal problem we consider is of the form

(13) $$\begin{array}{c} \underset{x\in X}{\min}\;F\left(\;Kx\;\right)+G\left(\;x\;\right), \end{array}$$

where *F* : *Y* → [0, +*∞* and *G* : *X* → [0, +*∞* are proper, convex, and lower semicontinuous and the map *K* : *X* → *Y* is a continuous linear operator. The corresponding primal-dual formulation of (13) is the saddle-point problem

(14) $$\begin{array}{c} \underset{x\in X}{\min}\;\underset{y\in Y}{\max}{\langle \;Kx,y\;\rangle }_{Y}+G\left(\;x\;\right)-F^{*}\left(\;y\;\right), \end{array}$$

with *F* ^*∗*^ : *Y* → *ℝ* ∪ {+*∞*} being the convex conjugate of *F*. We assume that the problems above have at least one solution $(\hat{x},\hat{y})\in X\times Y$ and therefore the following holds:

(15) Kx^∈∂F∗y^,−K∗y^∈∂Gx^,

where $\partial F^{*}(\hat{y})$ and $\partial G(\hat{x})$ are the subdifferentials of the convex functions *F* ^*∗*^ at $\hat{y}$ and *G* at $\hat{x}$ . Furthermore we assume that *F* and *G* are “simple”; that is, the resolvent operators (*I* + *σ*∂*F* ^*∗*^)^−1^ and (*I* + *τ*∂*G*)^−1^ are easy to compute. For a convex function *f* the resolvent of the operator *τ*∂*f* at $\tilde{x}$ can be calculated in our case by

(16) $$\begin{array}{c} x={\left(\;I+\tau \partial f\;\right)}^{-1}\left(\;\tilde{x}\;\right)=\underset{x}{\arg\min}\left\{\;\frac{{\left\|x-\tilde{x}\right\|}^{2}}{2\tau }+f\left(\;x\;\right)\;\right\}. \end{array}$$

In this paper we will only make use of Algorithm  1 in [17] with the extrapolation parameter *θ* = 1. Although interesting, the usage of the other proposed algorithms is left for the moment for later research.

To apply Algorithm  1 in [17] to the minimization problems (10), (11), and (12), we first need to rewrite them in their discretized version, then identify the functions *F* and *G*, and finally derive the resolvent operators (*I* + *σ*∂*F* ^*∗*^)^−1^ and (*I* + *τ*∂*G*)^−1^.

For the discrete setting we therefore define by

(17) xi,j=x1 i,j,x2 i,j=ih,jh,i=1,…,M,  j=1,…,N,

the pixel positions in the image domain with *h* being the spatial step size. For the calculations of the finite differences, the discrete divergence operator, the discretized inner products, and further details, we refer the reader to [17] and the references therein.

In the following sections we will derive the resolvent operators for the three given minimization problems (10), (11), and (12).

### 3.3. Resolvent Operators for Problem (10)

Let us consider the continuous problem (10). As mentioned already before, we need to rewrite the problem in its discretized version to be able to apply a primal-dual approach for the minimization. We use the spaces *X* = *ℝ* ^*MN*^ and *Y* = *X* × *X* and their inner products:

(18) s,tX=∑i,jsi,jti,j,s,t∈X,

(19) p,qY=∑i,jpi,j1qi,j1+pi,j2qi,j2,p=p1,p2,  q=q1,q2∈Y.

Using the rectangle rule we can rewrite problem (10) in the discretized version

(20) h2minu~∈0,1MN⁡ν∇u~1+∑i,jDwi,j+u~i,j+Dwi,j−1−u~i,j,

where the factor *h* ^2^ can be neglected, since it has no influence on the optimal solution. The norm ‖∇*s*‖_1_, with *s* ∈ *X*, is defined by ‖∇*s*‖_1_ = ∑_*i*,*j*_|(∇*s*)_*i*,*j*_|  , where $\left|{\left(\nabla s\right)}_{i,j}\right|=\sqrt{{\left({\left(\nabla s\right)}_{i,j}^{\left(1\right)}\right)}^{2}+{\left({\left(\nabla s\right)}_{i,j}^{\left(2\right)}\right)}^{2}}$ .

Comparing (20) to (13), we see that *K* = ∇, $F(\nabla \tilde{u})=\nu {\left\|\nabla \tilde{u}\right\|}_{1}$ , and $G(\tilde{u})=\sum_{i,j}D(w_{i,j}^{+})\;{\tilde{u}}_{i,j}+D(w_{i,j}^{-})(1-{\tilde{u}}_{i,j})$ . The solution of the resolvent operator with respect to *F* ^*∗*^ can be derived as

(21) p=I+σ∂F∗−1p~⟹pi,j=νp~i,jmax⁡ν,p~i,j

and the one with respect to *G* as

(22) u~=I+τ∂G−1u^⟹u~i,j=min⁡max⁡u^i,j−τDwi,j+−Dwi,j−,0,1.

**Remark 2 .**

The primal-dual formulation of problem (20) is a special case of the general problem considered in Pock et al.'s work [23] and the resulting primal-dual algorithm is then the same as described there. Namely, the dual variable *p* is projected onto the convex set, a disc with radius *ν*, and with a truncation of the primal variable $\tilde{u}$ the projection on the feasible convex set [0,1] is achieved.

**Remark 3 .**

Observant readers probably noticed that we slightly misused the mathematical notation to make the formulas more pleasing to the eye. More precisely, instead of writing *D*(**w**_*i*,*j*_^±^) we should have been using *D*(**w** ^±^)|_*i*,*j*_, since discretization is performed after the function *D* is applied. In the following we will also make use of this sloppy notation for the functions *ρ*_1_, *ρ*_2_^(1)^, and *ρ*_2_^(2)^ given in (9).

### 3.4. Resolvent Operators for Problem (11)

Now we consider the continuous problem (11). If we have a closer look at this minimization problem, we see that we only receive information about **w** ^+^ on the domain Σ where $\tilde{u}$ will be set to 1. Although theoretically reasonable, for the numerical calculations the implementation gets facilitated by having a smooth extension of **w** ^+^ to the domain *Ω*∖Σ. We therefore consider instead the problem

(23) $$\begin{array}{c} \underset{w^{+}}{\min}\left\{\;\int_{\Omega }f\left(\;w^{+}\;\right)\tilde{u}\left(\;x\;\right)+\mu s\left(\;w^{+}\;\right)dx\;\right\}. \end{array}$$

Comparing (11) to (23) the only difference is that the factor $\tilde{u}$ is not applied to the smoothness term *s* anymore. For the primal-dual approach we will use this time the spaces *X* = *ℝ* ^*MN*^ × *ℝ* ^*MN*^ and *Y* = *X* × *X*. The inner product of *X* is the same as in (19) and the one for *Y* is defined by

(24) p,qY=∑i,jpi,j1qi,j1+pi,j2qi,j2+pi,j3qi,j3+pi,j4qi,j4,p=p1,p2,p3,p4,  q=q1,q2,q3,q4∈Y.

After the discretization of problem (23) we obtain

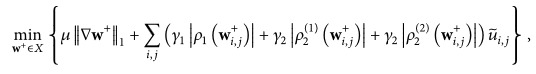

(25)
where this time the norm ‖∇*s*‖_1_, with *s* = (*s*_1_, *s*_2_) ∈ *X*, is defined by ‖∇*s*‖_1_ = ∑_*i*,*j*_|(∇*s*)_*i*,*j*_|  with $\left|{\left(\nabla s\right)}_{i,j}\right|=\sqrt{{\left({\left(\nabla s_{1}\right)}_{i,j}^{\left(1\right)}\right)}^{2}+{\left({\left(\nabla s_{1}\right)}_{i,j}^{\left(2\right)}\right)}^{2}+{\left({\left(\nabla s_{2}\right)}_{i,j}^{\left(1\right)}\right)}^{2}+{\left({\left(\nabla s_{2}\right)}_{i,j}^{\left(2\right)}\right)}^{2}}$ .

Comparing (25) to (13), we see that the corresponding functions are *K* = ∇, *F*(∇**w** ^+^) = *μ*‖∇**w** ^+^‖_1_, and

(26) Gw+=∑i,jγ1ρ1wi,j++γ2ρ21wi,j++γ2ρ22wi,j+u~i,j.

From the resolvent operator with respect to *F* ^*∗*^ we obtain

(27) q=I+σ∂F∗−1q~⟹qi,j=μq~i,jmax⁡μ,q~i,j.

The derivation of the resolvent operator with respect to *G* is not that straightforward and more effort has to be put in to find a suitable solution. It is common that the resolvent operators of functions, which are sums of quadratic and absolute norms, lead to so-called thresholding schemes [24, 25]. This will be also the case here. Having a closer look at the definition of *G* (26) and (16), we see that we have to solve

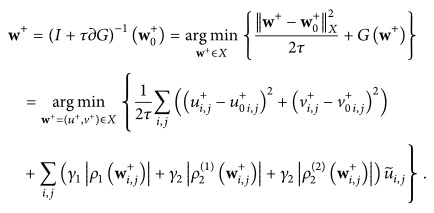

(28)
For ${\tilde{u}}_{i,j}=0$ we can conclude from (28) that **w**_*i*,*j*_^+^ = **w**_0 *i*,*j*_^+^. On the other hand, for ${\tilde{u}}_{i,j}=1$ we have to distinguish the cases

(29) $$\begin{array}{c} \rho_{1}\left(\;w_{i,j}^{+}\;\right)\left(\begin{array}{c} >\\ <\\ = \end{array}\right)0,\\ \rho_{2}^{\left(1\right)}\left(\;w_{i,j}^{+}\;\right)\left(\begin{array}{c} >\\ <\\ = \end{array}\right)0,\\ \rho_{2}^{\left(2\right)}\left(\;w_{i,j}^{+}\;\right)\left(\begin{array}{c} >\\ <\\ = \end{array}\right)0, \end{array}$$

which turn out to be 27 in total. The chosen numbering for the different cases is shown in Table 1.

#### 3.4.1. Geometric Interpretation of Problem (28) with ${\tilde{u}}_{i,j}=1$

Before explaining the derivation of the explicit solutions and the needed reformulation of the conditions for the different cases in (29) in more detail, we want to give a deeper insight of the geometric interpretation of problem (28) with ${\tilde{u}}_{i,j}=1$ . To facilitate the notation in the following, we introduce the terms

(30) ai,j=∇Tx+w0+i,j,bi,j=∂x1x1Tx+w0+∂x2x1Tx+w0+i,j,ci,j=∂x1x2Tx+w0+∂x2x2Tx+w0+i,j,

(31) xi,j=τγ1ai,j,yi,j=τγ2bi,j,zi,j=τγ2ci,j.

Using the definitions in (9) and the notation in (30), we can rewrite *ρ*_1_(**w**_*i*,*j*_^+^), *ρ*_2_^(1)^(**w**_*i*,*j*_^+^), and *ρ*_2_^(2)^(**w**_*i*,*j*_^+^) as

(32) ρ1wi,j+=ρ1w0 i,j++ai,jTwi,j+−w0 i,j+,ρ21wi,j+=ρ21w0 i,j++bi,jTwi,j+−w0 i,j+,ρ22wi,j+=ρ22w0 i,j++ci,jTwi,j+−w0 i,j+.

Note that with *ρ*_1_(**w**_*i*,*j*_^+^) = 0 a line *l*_*a*_*i*,*j*__ is defined with its normal vector being parallel to the vector *a*_*i*,*j*_. Similarly, *ρ*_2_^(1)^(**w**_*i*,*j*_^+^) = 0 defines a line *l*_*b*_*i*,*j*__ and *ρ*_2_^(2)^(**w**_*i*,*j*_^+^) = 0 a line *l*_*c*_*i*,*j*__.

**Figure 2:**
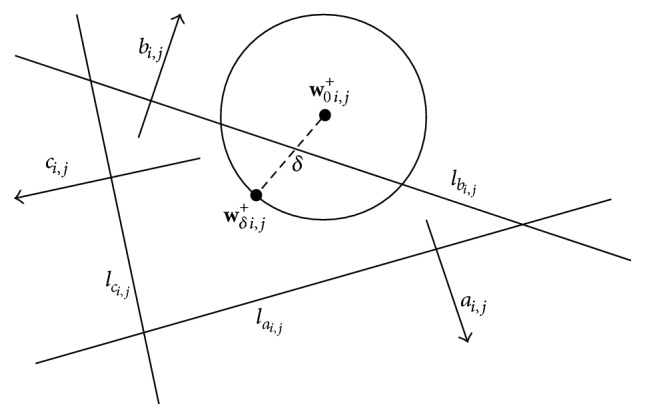
The geometric interpretation of minimization problem (28) with ${\tilde{u}}_{i,j}=1$ .

Now we can argue similar to Zach et al. in [25] for the geometric interpretation. The mathematical structure of their minimization problem for which they derive the thresholding scheme is very similar to the one we have in (28) with ${\tilde{u}}_{i,j}=1$ . The first term in (28), (*u*_*i*,*j*_^+^ − *u*_0 *i*,*j*_^+^)^2^ + (*v*_*i*,*j*_^+^ − *v*_0 *i*,*j*_^+^)^2^, is the squared distance of **w**_*i*,*j*_^+^ to **w**_0 *i*,*j*_^+^, and the terms |*ρ*_1_(**w**_*i*,*j*_^+^)|, |*ρ*_2_^(1)^(**w**_*i*,*j*_^+^)|, and |*ρ*_2_^(2)^(**w**_*i*,*j*_^+^)| define the unsigned distances to the lines *l*_*a*_*i*,*j*__, *l*_*b*_*i*,*j*__, and *l*_*c*_*i*,*j*__, respectively. Considering now all **w**_*δ* *i*,*j*_^+^ with a fixed distance *δ* to **w**_0 *i*,*j*_^+^, we see that problem (28) with ${\tilde{u}}_{i,j}=1$ is minimized for **w**_*δ* *i*,*j*_^+^ closest to the three lines *l*_*a*_*i*,*j*__, *l*_*b*_*i*,*j*__, and *l*_*c*_*i*,*j*__. See Figure 2 for an illustration.

#### 3.4.2. Derivation of the Explicit Solutions for Problem (28) with ${\tilde{u}}_{i,j}=1$

We are ready now to derive for each of the cases an explicit solution by using (28). We show the derivation of the solutions only for four different cases. The explicit solutions for the remaining cases can be calculated in a similar fashion as the presented ones.

*Case  1 and Similar Ones.* Let us consider the first case in Table 1; that is,

(33) ρ1wi,j+=ρ1w0 i,j++ai,jTwi,j+−w0 i,j+>0,ρ21wi,j+=ρ21w0 i,j++bi,jTwi,j+−w0 i,j+>0,ρ22wi,j+=ρ22w0 i,j++ci,jTwi,j+−w0 i,j+>0.

Solving problem (28) for this case leads to the equation

(34) $$\begin{array}{c} \frac{1}{2\tau }2\left(\;w_{i,j}^{+}-w_{0\;i,j}^{+}\;\right)+\gamma_{1}a_{i,j}+\gamma_{2}b_{i,j}+\gamma_{2}c_{i,j}\overset{!}{=}0, \end{array}$$

and by using the notation in (31) the explicit solution can then be written as

(35) $$\begin{array}{c} w_{i,j}^{+}=w_{0\;i,j}^{+}-x_{i,j}-y_{i,j}-z_{i,j}. \end{array}$$

The derivation of the explicit solutions for cases 2, 4, 5, 7, 8, 10, and 11 in Table 1 is performed similarly. There are however other cases left which need another treatment, as, for example, case number 3.

*Case  3 and Similar Ones.* The condition for case 3 in Table 1 is given by

(36) ρ1wi,j+=ρ1w0 i,j++ai,jTwi,j+−w0 i,j+=0,ρ21wi,j+=ρ21w0 i,j++bi,jTwi,j+−w0 i,j+>0,ρ22wi,j+=ρ22w0 i,j++ci,jTwi,j+−w0 i,j+>0.

Here we have to distinguish the two situations *a*_*i*,*j*_ ≠ 0 and *a*_*i*,*j*_ = 0.

(i) If *a*_*i*,*j*_ ≠ 0, we have the following.

From *ρ*_1_(**w**_*i*,*j*_^+^) = 0 we see that the solution **w**_*i*,*j*_^+^ has to lie on the line *l*_*a*_*i*,*j*__ and of course the distance of it to the other lines *l*_*b*_*i*,*j*__ and *l*_*c*_*i*,*j*__ should be minimal. Therefore we can assume that the solution is of the form

(37) $$\begin{array}{c} w_{i,j}^{+}=w_{0\;i,j}^{+}+\alpha_{1}a_{i,j}+\beta_{0pp}n_{a_{i,j}}, \end{array}$$

where *n*_*a*_*i*,*j*__ is the normal to *a*_*i*,*j*_. Geometrically this means that **w**_0 *i*,*j*_^+^ is first orthogonally projected to the line *l*_*a*_*i*,*j*__ and afterwards moved along the line such that it minimizes the distance to *l*_*b*_*i*,*j*__ and *l*_*c*_*i*,*j*__. The unknown *α*_1_ is determined by replacing the term (**w**_*i*,*j*_^+^ − **w**_0 *i*,*j*_^+^) in the equation *ρ*_1_(**w**_*i*,*j*_^+^) = 0 using (37), which leads to

(38) $$\begin{array}{c} \alpha_{1}=-\frac{\rho_{1}\left(w_{0\;i,j}^{+}\right)}{{\left|a_{i,j}\right|}^{2}}. \end{array}$$

Solving problem (28) for the current case and replacing again the term (**w**_*i*,*j*_^+^ − **w**_0 *i*,*j*_^+^) using (37) lead after some simple calculations to the second parameter

(39) $$\begin{array}{c} \beta_{0pp}=\frac{n_{a_{i,j}}^{T}\left(-y_{i,j}-z_{i,j}\right)}{{\left|n_{a_{i,j}}\right|}^{2}}. \end{array}$$

(ii) If *a*_*i*,*j*_ = 0, we have the following.

Since *a*_*i*,*j*_ vanishes, there is also no line *l*_*a*_*i*,*j*__ that we have to consider and we can directly derive the solution **w**_*i*,*j*_^+^ by just solving (28), similar to the first case in Table 1, and we get the solution

(40) $$\begin{array}{c} w_{i,j}^{+}=w_{0\;i,j}^{+}-y_{i,j}-z_{i,j}. \end{array}$$

Finally, we can derive the explicit solution for cases 6, 9, 12, 13, 14, 16, 17, 22, 23, 25, and 26 in Table 1 similar to case 3 above.

*Case  15 and Similar Ones.* Let us now consider case number 15, which was not covered so far. The condition is this time given by

(41) ρ1wi,j+=ρ1w0 i,j++ai,jTwi,j+−w0 i,j+=0,ρ21wi,j+=ρ21w0 i,j++bi,jTwi,j+−w0 i,j+=0,ρ22wi,j+=ρ22w0 i,j++ci,jTwi,j+−w0 i,j+>0,

and we see that we have now two equal signs, namely, for *ρ*_1_(**w**_*i*,*j*_^+^) and *ρ*_2_^(1)^(**w**_*i*,*j*_^+^). This time we have to distinguish the situations *a*_*i*,*j*_ ≠ 0, *a*_*i*,*j*_ = 0∧*b*_*i*,*j*_ ≠ 0, and *a*_*i*,*j*_ = 0∧*b*_*i*,*j*_ = 0.

(i) If *a*_*i*,*j*_ ≠ 0, we have the following.

From *ρ*_1_(**w**_*i*,*j*_^+^) = 0 and *ρ*_2_^(1)^(**w**_*i*,*j*_^+^) = 0 we see that the solution **w**_*i*,*j*_^+^ has to lie on the lines *l*_*a*_*i*,*j*__ and *l*_*b*_*i*,*j*__ and should have a minimal distance to the line *l*_*c*_*i*,*j*__. Thus, we assume that the explicit solution is of the form

(42) $$\begin{array}{c} w_{i,j}^{+}=w_{0\;i,j}^{+}+\alpha_{1}a_{i,j}+\beta_{00p}n_{a_{i,j}}. \end{array}$$

Similar to the last paragraph, replacing the term (**w**_*i*,*j*_^+^ − **w**_0 *i*,*j*_^+^) in the equation *ρ*_1_(**w**_*i*,*j*_^+^) = 0 by making use of (42) leads to the same *α*_1_ as in (38). For the parameter *β*_00*p*_ we get

(43) $$\begin{array}{c} \beta_{00p}=\left\{\begin{array}{cc} \frac{-\rho_{2}^{\left(1\right)}\left(w_{0\;i,j}^{+}\right)-\alpha_{1}b_{i,j}^{T}a_{i,j}}{b_{i,j}^{T}n_{a_{i,j}}}, & \text{if }b_{i,j}^{T}n_{a_{i,j}}\neq 0,\\ \frac{n_{a_{i,j}}^{T}\left(-z_{i,j}\right)}{{\left|n_{a_{i,j}}\right|}^{2}}, & \text{if }b_{i,j}^{T}n_{a_{i,j}}=0. \end{array}\right. \end{array}$$

The case where *b*_*i*,*j*_^T^ *n*_*a*_*i*,*j*__ ≠ 0 in (43), which is equivalent to *a*_*i*,*j*_∦*b*_*i*,*j*_, indicates that the lines *l*_*a*_*i*,*j*__ and *l*_*b*_*i*,*j*__ are not parallel and that they therefore intersect each other in one point. The solution **w**_*i*,*j*_^+^ we look for will be at this intersection and the corresponding *β*_00*p*_ is calculated by replacing the term (**w**_*i*,*j*_^+^ − **w**_0 *i*,*j*_^+^) in the equation *ρ*_2_^(1)^(**w**_*i*,*j*_^+^) = 0 using (42). For the case where *b*_*i*,*j*_^T^ *n*_*a*_*i*,*j*__ = 0 in (43), which is equivalent to *a*_*i*,*j*_||*b*_*i*,*j*_, we conclude that the lines *l*_*a*_*i*,*j*__ and *l*_*b*_*i*,*j*__ are parallel. This means that if the solution **w**_*i*,*j*_^+^ lies on the line *l*_*a*_*i*,*j*__ it necessarily also lies on the line *l*_*b*_*i*,*j*__ and the parameter *β*_00*p*_ is calculated by solving problem (28) for the current case and replacing the term (**w**_*i*,*j*_^+^ − **w**_0 *i*,*j*_^+^) using (42).

(ii) If *a*_*i*,*j*_ = 0∧*b*_*i*,*j*_ ≠ 0, we have the following.

Since *a*_*i*,*j*_ vanishes, there is no need to consider the line *l*_*a*_*i*,*j*__ and we focus instead on the line *l*_*b*_*i*,*j*__ on which the solution **w**_*i*,*j*_^+^ has to lie too. We therefore assume the solution has the form

(44) $$\begin{array}{c} w_{i,j}^{+}=w_{0\;i,j}^{+}+\alpha_{2}b_{i,j}+\beta_{00p}n_{b_{i,j}}. \end{array}$$

Replacing this time the term (**w**_*i*,*j*_^+^ − **w**_0 *i*,*j*_^+^) in the equation *ρ*_2_^(1)^(**w**_*i*,*j*_^+^) = 0 with the help of (44) results in

(45) $$\begin{array}{c} \alpha_{2}=-\frac{\rho_{2}^{\left(1\right)}\left(w_{0\;i,j}^{+}\right)}{{\left|b_{i,j}\right|}^{2}}. \end{array}$$

After solving problem (28) and replacing the term (**w**_*i*,*j*_^+^ − **w**_0 *i*,*j*_^+^) using (44) we get

(46) $$\begin{array}{c} \beta_{00p}=\frac{n_{b_{i,j}}^{T}\left(-z_{i,j}\right)}{{\left|n_{b_{i,j}}\right|}^{2}}. \end{array}$$

(iii) If *a*_*i*,*j*_ = 0∧*b*_*i*,*j*_ = 0, we have the following.

This time both *a*_*i*,*j*_ and *b*_*i*,*j*_ vanish and the corresponding lines *l*_*a*_*i*,*j*__ and *l*_*b*_*i*,*j*__ do not play a role in the derivation of the solution **w**_*i*,*j*_^+^ anymore. We can directly solve problem (28) without any prior assumption, similarly as done before in other cases, and we get

(47) $$\begin{array}{c} w_{i,j}^{+}=w_{0\;i,j}^{+}-z_{i,j}. \end{array}$$

The derivation of the explicit solutions for cases 18, 19, 20, 24, and 27 in Table 1 is done similarly to case 15 explained above. 

*Case  21.* There is now one case left, which was not discussed so far. The corresponding condition from Table 1 is

(48) ρ1wi,j+=ρ1w0 i,j++ai,jTwi,j+−w0 i,j+=0,ρ21wi,j+=ρ21w0 i,j++bi,jTwi,j+−w0 i,j+=0,ρ22wi,j+=ρ22w0 i,j++ci,jTwi,j+−w0 i,j+=0.

This time we have three equal signs and we have to distinguish the situations *a*_*i*,*j*_ ≠ 0, *a*_*i*,*j*_ = 0∧*b*_*i*,*j*_ ≠ 0, *a*_*i*,*j*_ = 0∧*b*_*i*,*j*_ = 0∧*c*_*i*,*j*_ ≠ 0, and *a*_*i*,*j*_ = 0∧*b*_*i*,*j*_ = 0∧*c*_*i*,*j*_ = 0.

(i) If *a*_*i*,*j*_ ≠ 0, we have the following.

Since *ρ*_1_(**w**_*i*,*j*_^+^) = 0, *ρ*_2_^(1)^(**w**_*i*,*j*_^+^) = 0, and *ρ*_2_^(2)^(**w**_*i*,*j*_^+^) = 0, we see that the solution **w**_*i*,*j*_^+^ has to lie this time on all three lines *l*_*a*_*i*,*j*__, *l*_*b*_*i*,*j*__, and *l*_*c*_*i*,*j*__. For *a*_*i*,*j*_ ≠ 0 we assume that the explicit solution is of the form

(49) $$\begin{array}{c} w_{i,j}^{+}=w_{0\;i,j}^{+}+\alpha_{1}a_{i,j}+\beta_{000}n_{a_{i,j}}. \end{array}$$

Like in the paragraphs before, replacing the term (**w**_*i*,*j*_^+^ − **w**_0 *i*,*j*_^+^) in *ρ*_1_(**w**_*i*,*j*_^+^) = 0 by making use of (49) leads to *α*_1_ being equal to the one in (38). To determine *β*_000_ in (49), we argue as follows:

(a) If *b*_*i*,*j*_^T^ *n*_*a*_*i*,*j*__ ≠ 0, which is equivalent to *a*_*i*,*j*_∦*b*_*i*,*j*_, we use (49) in *ρ*_2_^(1)^(**w**_*i*,*j*_^+^) = 0 and get
(50) $$\begin{array}{c} \beta_{000}=\frac{-\rho_{2}^{\left(1\right)}\left(w_{0\;i,j}^{+}\right)-\alpha_{1}b_{i,j}^{T}a_{i,j}}{b_{i,j}^{T}n_{a_{i,j}}}. \end{array}$$
(b) If *b*_*i*,*j*_^T^ *n*_*a*_*i*,*j*__ = 0 and *c*_*i*,*j*_^T^ *n*_*a*_*i*,*j*__ ≠ 0, that is, *a*_*i*,*j*_||*b*_*i*,*j*_ and *a*_*i*,*j*_∦*c*_*i*,*j*_, we use (49) in *ρ*_2_^(2)^(**w**_*i*,*j*_^+^) = 0 and get
(51) $$\begin{array}{c} \beta_{000}=\frac{-\rho_{2}^{\left(2\right)}\left(w_{0\;i,j}^{+}\right)-\alpha_{1}c_{i,j}^{T}a_{i,j}}{c_{i,j}^{T}n_{a_{i,j}}}. \end{array}$$
(c) If *b*_*i*,*j*_^T^ *n*_*a*_*i*,*j*__ = 0 and *c*_*i*,*j*_^T^ *n*_*a*_*i*,*j*__ = 0, this means that *a*_*i*,*j*_||*b*_*i*,*j*_ and *a*_*i*,*j*_||*c*_*i*,*j*_. Thus all the three lines *l*_*a*_*i*,*j*__, *l*_*b*_*i*,*j*__, and *l*_*c*_*i*,*j*__ are parallel and by solving problem (28) and using (49) we get
(52) $$\begin{array}{c} \beta_{000}=0. \end{array}$$
(ii) If *a*_*i*,*j*_ = 0∧*b*_*i*,*j*_ ≠ 0, we have the following.

Similarly as in the discussion of (44) in case 15, we assume the solution is of the form

(53) $$\begin{array}{c} w_{i,j}^{+}=w_{0\;i,j}^{+}+\alpha_{2}b_{i,j}+\beta_{000}n_{b_{i,j}}. \end{array}$$

Replacing the term (**w**_*i*,*j*_^+^ − **w**_0 *i*,*j*_^+^) in *ρ*_2_^(1)^(**w**_*i*,*j*_^+^) = 0 by making use of (53) leads to *α*_2_ being equal to the one in (45). For *β*_000_ in (53), we argue as follows:

(a) If *c*_*i*,*j*_^T^ *n*_*b*_*i*,*j*__ ≠ 0, which is equivalent to *b*_*i*,*j*_∦*c*_*i*,*j*_, we make use of (53) in *ρ*_2_^(2)^(**w**_*i*,*j*_^+^) = 0 and get
(54) $$\begin{array}{c} \beta_{000}=\frac{-\rho_{2}^{\left(2\right)}\left(w_{0\;i,j}^{+}\right)-\alpha_{2}c_{i,j}^{T}b_{i,j}}{c_{i,j}^{T}n_{b_{i,j}}}. \end{array}$$
(b) If *c*_*i*,*j*_^T^ *n*_*b*_*i*,*j*__ = 0, which means *b*_*i*,*j*_||*c*_*i*,*j*_, we get by solving (28) and using (53)
(55) $$\begin{array}{c} \beta_{000}=0. \end{array}$$
(iii) If *a*_*i*,*j*_ = 0∧*b*_*i*,*j*_ = 0∧*c*_*i*,*j*_ ≠ 0, we have the following.

Here, *a*_*i*,*j*_ and *b*_*i*,*j*_ vanish and we focus therefore on the line *l*_*c*_*i*,*j*__ on which the solution **w**_*i*,*j*_^+^ has to lie. We assume the explicit solution is of the form

(56) $$\begin{array}{c} w_{i,j}^{+}=w_{0\;i,j}^{+}+\alpha_{3}c_{i,j}, \end{array}$$

where *α*_3_ is calculated by using (56) to replace (**w**_*i*,*j*_^+^ − **w**_0 *i*,*j*_^+^) in *ρ*_2_^(2)^(**w**_*i*,*j*_^+^) = 0 and is given by

(57) $$\begin{array}{c} \alpha_{3}=-\frac{\rho_{2}^{\left(2\right)}\left(w_{0\;i,j}^{+}\right)}{{\left|c_{i,j}\right|}^{2}}. \end{array}$$

(iv) If *a*_*i*,*j*_ = 0∧*b*_*i*,*j*_ = 0∧*c*_*i*,*j*_ = 0, we have the following.

In this situation we do not have to consider the lines *l*_*a*_*i*,*j*__, *l*_*b*_*i*,*j*__, and *l*_*c*_*i*,*j*__ at all and solving (28) results in

(58) $$\begin{array}{c} w_{i,j}^{+}=w_{0\;i,j}^{+}. \end{array}$$

#### 3.4.3. Reformulation of the Conditions for Problem (28) with ${\tilde{u}}_{i,j}=1$

**Figure 3:**
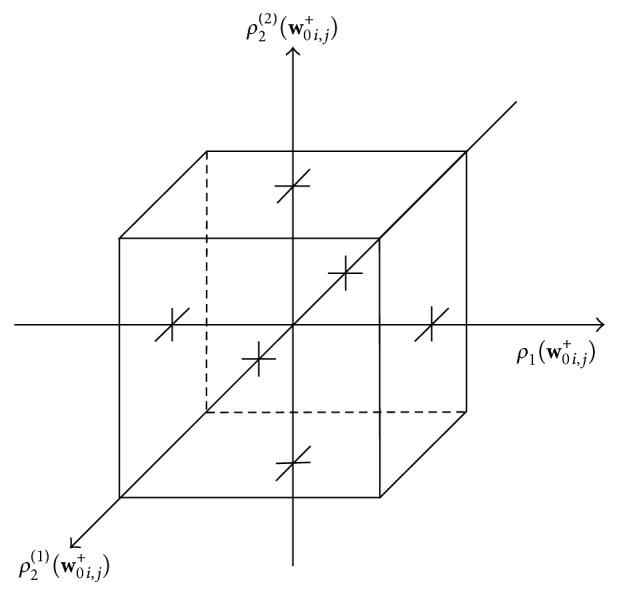
The space spanned by *ρ*_1_(**w**_0 *i*,*j*_^+^), *ρ*_2_^(1)^(**w**_0 *i*,*j*_^+^), and *ρ*_2_^(2)^(**w**_0 *i*,*j*_^+^).

Since **w**_*i*,*j*_^+^ is unknown but **w**_0 *i*,*j*_^+^ in (9) is known, we have to rewrite the conditions for the 27 cases, which are defined by (29), in terms of *ρ*_1_(**w**_0 *i*,*j*_^+^), *ρ*_2_^(1)^(**w**_0 *i*,*j*_^+^), and *ρ*_2_^(2)^(**w**_0 *i*,*j*_^+^). This can be done by incorporating the derived explicit solutions and finding proper partitioning of the space spanned by *ρ*_1_(**w**_0 *i*,*j*_^+^), *ρ*_2_^(1)^(**w**_0 *i*,*j*_^+^), and *ρ*_2_^(2)^(**w**_0 *i*,*j*_^+^) (see Figure 3). A similar approach was used in the work of Zach et al. [25] to reformulate the conditions for their proposed thresholding scheme.

**Figure 4:**
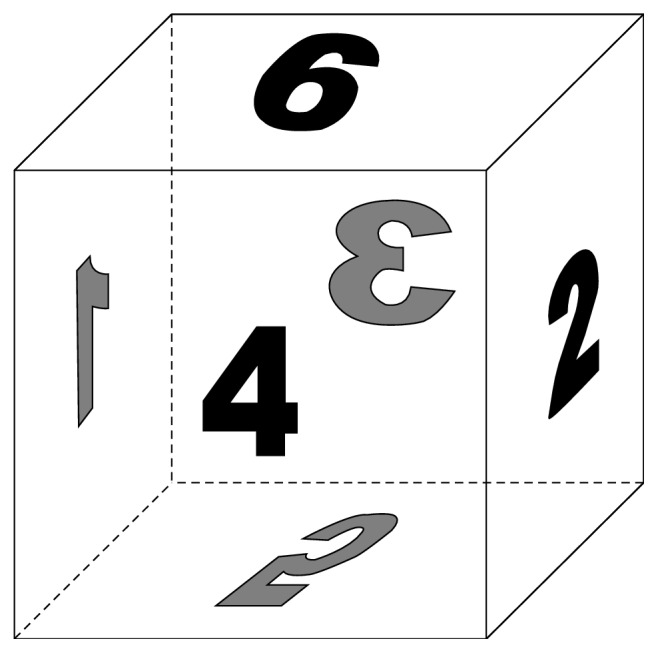
The numbering of the faces for the cube indicated in Figure 3.

As one can see in Figure 3, we indicated a cube in the coordinate system that will facilitate for us the problem of finding proper partitioning of the considered space. The edge length of the cube is chosen in such a way that the critical values of the reformulated conditions are covered. The reader will understand the meaning of this sentence afterwards in a better way. For later need, we number the faces of the cube as shown in Figure 4.

The orientation of the face numbers in Figure 4 indicates from which direction we will look at the faces.

In the following we want to show how we can rewrite the conditions for the different cases. The same four groups of cases which were discussed in Section 3.4.2 are considered here too and for each of them the reformulation of the conditions can be achieved in a similar fashion. 

*Case  1 and Similar Ones.* We start again with the first case in Table 1, which belongs to the group of cases that can be handled in the easiest way compared to the other ones. The condition for this case is given in (33) and by using (35) to replace the term (**w**_*i*,*j*_^+^ − **w**_0 *i*,*j*_^+^) in the condition we get

(59) ρ1wi,j+=ρ1w0 i,j++ai,jT−xi,j−yi,j−zi,j>0,ρ21wi,j+=ρ21w0 i,j++bi,jT−xi,j−yi,j−zi,j>0,ρ22wi,j+=ρ22w0 i,j++ci,jT−xi,j−yi,j−zi,j>0,

or rewritten

(60) ρ1w0 i,j+>−ai,jT−xi,j−yi,j−zi,j,ρ21w0 i,j+>−bi,jT−xi,j−yi,j−zi,j,ρ22w0 i,j+>−ci,jT−xi,j−yi,j−zi,j.

One of the critical values we mentioned before concerning the edge length of the cube shown in Figure 3 can be determined now for case number 1, namely, by the values on the right-hand side in (60).

The reformulation of the conditions for cases 2, 4, 5, 7, 8, 10, and 11 in Table 1 is achieved similarly to this case and the remaining seven critical values, which are necessary to define the edge length of the cube, are determined in the same way as above.

**Remark 4 .**

**Figure 5:**
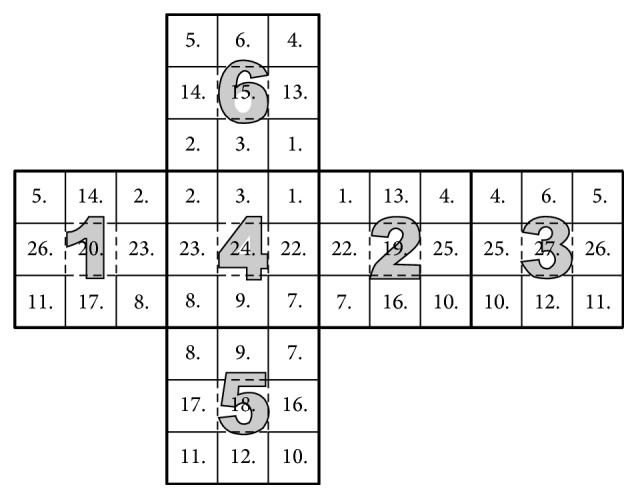
The net of the cube indicated in Figure 3 with the corresponding case numbers for the different regions. The face numbers from Figure 4 are visible in the background.

Before jumping to the next group of cases, we want to explain why the cube in Figure 3 will help us in finding proper partitioning of the space spanned by *ρ*_1_(**w**_0 *i*,*j*_^+^), *ρ*_2_^(1)^(**w**_0 *i*,*j*_^+^), and *ρ*_2_^(2)^(**w**_0 *i*,*j*_^+^). Our aim is to divide the space into 27 parts, each one defining a region for which exactly one of the 27 cases may apply with their already derived explicit solutions. This partitioning should not be done just arbitrarily and has to be meaningful and to conform to the already set up formulations. Before, we mentioned once that we want the eight critical values from the paragraph before to be covered by the cube. The reason for this is that like this it is possible to reduce the partitioning problem of the whole space to a partitioning problem of the cube. The uniquely defined regions of the cube are the ones from the corresponding eight cases discussed in the paragraph above and they define the corners of it. In Figure 5 we show the location of the regions for different case numbers. For simplicity we drew all the regions with the same size, although they can and most likely will have different sizes. Note that the region for case number 21 is not visible in the figure, because it fully lies in the interior of the cube.

**Figure 6:**
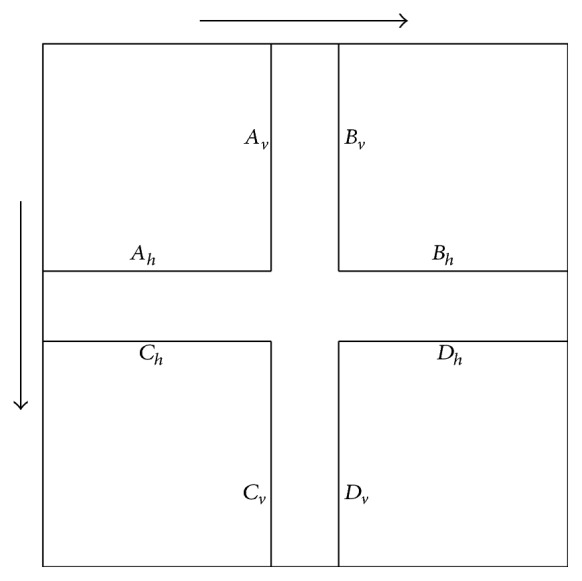
One face of the cube with the depicted corner regions and their labelling for the boundary values. The arrows indicate the direction of increasement.

Let us focus now on one face of the cube. We know the values for the boundaries of the corner regions from the critical values we got from the previous paragraph. They are fixed and we introduce for them the names *A*_*v*_, *A*_*h*_, *B*_*v*_, *B*_*h*_, *C*_*v*_, *C*_*h*_, *D*_*v*_, and *D*_*h*_ as shown in Figure 6.

For face number 6, for example, the values *D*_*v*_ and *D*_*h*_ will be the boundary values to region 1 (see Figure 5) and after having a look at (60) we can conclude that *D*_*v*_ = −*a*_*i*,*j*_^T^(−*x*_*i*,*j*_ − *y*_*i*,*j*_ − *z*_*i*,*j*_) and *D*_*h*_ = −*b*_*i*,*j*_^T^(−*x*_*i*,*j*_ − *y*_*i*,*j*_ − *z*_*i*,*j*_).

For each face it always holds that

(61) Av≤Bv,Cv≤Dv,Bh≤Dh,Ah≤Ch.

For example, to see that *C*_*v*_ ≤ *D*_*v*_ we consider the reformulation of the condition for case number 2:

(62) ρ1w0 i,j+<−ai,jT+xi,j−yi,j−zi,j,ρ21w0 i,j+>−bi,jT+xi,j−yi,j−zi,j,ρ22w0 i,j+>−ci,jT+xi,j−yi,j−zi,j,

which can be derived, as mentioned before, similarly to case number 1. For face number 6 we see in the same manner as before by having a look at Figure 5 and (62) that *C*_*v*_ = −*a*_*i*,*j*_^T^(+*x*_*i*,*j*_ − *y*_*i*,*j*_ − *z*_*i*,*j*_). Since *a*_*i*,*j*_^T^ *x*_*i*,*j*_ = *τγ*_1_|*a*_*i*,*j*_|^2^ ≥ 0 (see (31)), we can conclude that *C*_*v*_ ≤ *D*_*v*_.

**Figure 7:**
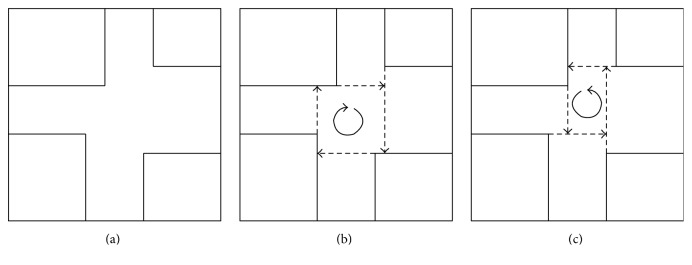
A face with its depicted corner regions (a) and the clockwise (b) and anticlockwise (c) division method, respectively.

Let us consider again an arbitrary face of the cube. Although we have boundaries for the corner regions, the ones for the regions in between are not fully defined. Since a rectangular division of the face seems to be adequate, we investigate the problem on how to achieve such one. In Figure 7 a face is depicted with its corner regions and two ways of possible divisions into 9 rectangles are shown, which we call the “clockwise” and “anticlockwise” division method.

Depending on the values for *A*_*v*_, *A*_*h*_, *B*_*v*_, *B*_*h*_, *C*_*v*_, *C*_*h*_, *D*_*v*_, and *D*_*h*_ sometimes both the clockwise and the anticlockwise division method work, as, for example, in Figure 7. Depending on the situation however, there can be also values for which only the clockwise or the anticlockwise or even none of both methods will work, where in the latter situation another adequate division method has to be defined.

In Table 2 we list the different possible situations for the values *A*_*v*_, *A*_*h*_, *B*_*v*_, *B*_*h*_, *C*_*v*_, *C*_*h*_, *D*_*v*_, and *D*_*h*_.

Furthermore, we split the last situation S9 into its parts for later use and introduce the corresponding labelling in Table 3.

**Figure 8:**
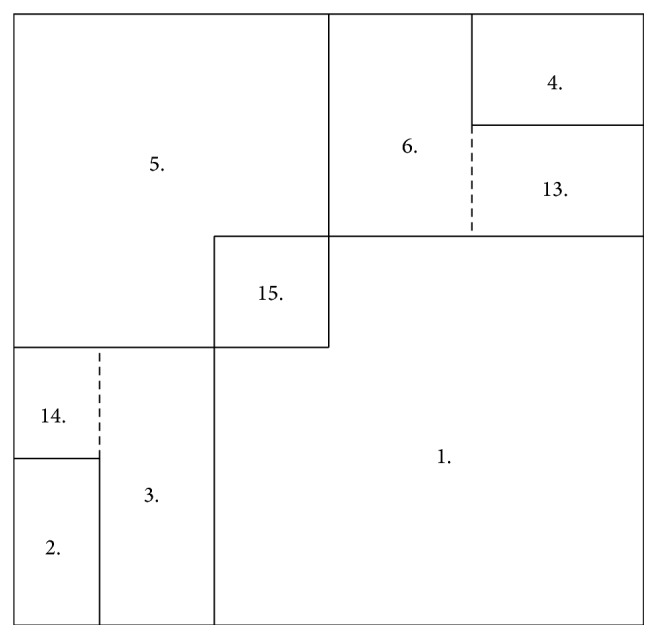
The division method used for situation S1 in Table 2. For the illustration we used face number 6 and the corresponding region numbers.

**Figure 9:**
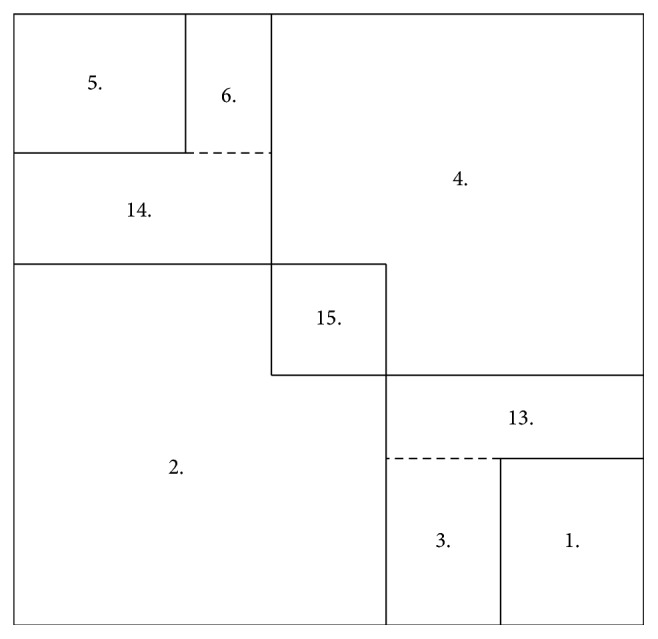
The division method used for situation S9.7 in Table 3. For the illustration we used again face number 6 and the corresponding region numbers.

In the following, we go through the different situations given in Tables 2 and 3 and decide which kind of method can be used to achieve a division of the face into 9 rectangular regions:

(i) Situation S1 in Table 2 is illustrated in Figure 8 for face number 6 and the corresponding region numbers. For this situation, neither the clockwise nor the anticlockwise division method will work, since the corner regions 1 and 5 overlap. An adapted division method, which is depicted in Figure 8, will be used instead for this situation. Note that in this situation certain corner regions, which were defined through the reformulation of the conditions for the group of cases similar to case 1, have to be adapted by removing from them the region where they overlap.
(ii) For the situations S2, S3, S5, and S6 in Table 2 and S9.1, S9.2, S9.3, S9.4, and S9.5 in Table 3 we can use the clockwise division method illustrated in Figure 7. We define the set of situations for which the clockwise division method can be applied by
(63) $$\begin{array}{c} S_{c}≔\left\{\;S2,S3,S5,S6,S9.1,S9.2,S9.3,S9.4,S9.5\;\right\}. \end{array}$$
(iii) Situation S9.7 in Table 3 needs again a special treatment to achieve a meaningful division, since neither the clockwise nor the anticlockwise division method can be applied because of the overlapping of the corner regions 2 and 4, if we consider face number 6, for example. The adapted division method, which will be used instead, is shown in Figure 9 for face number 6 and the corresponding region numbers. Note again that also in this situation certain corner regions have to be adapted by removing from them the region where they overlap.
(iv) Finally, for the situations S4, S7, and S8 in Table 2 and S9.6, S9.8, and S9.9 in Table 3 we can use this time the anticlockwise division method illustrated in Figure 7. The set of situations for which the anticlockwise division method can be applied is then given by
(64) $$\begin{array}{c} S_{ac}≔\left\{\;S4,S7,S8,S9.6,S9.8,S9.9\;\right\}. \end{array}$$

We are now able to give an idea on how to reformulate the conditions for the remaining group of cases.

*Case  3 and Similar Ones.* The reformulation of the cases in this group with the same approach as for case number 1 will not lead to proper partitioning of the space shown in Figure 3. Instead we will use a more geometrically based approach which depends on the different situations the boundary values can take.

First we can derive for sure, independently of which approach we use, that

(65) $$\begin{array}{c} \left|\;\rho_{1}\left(\;w_{0\;i,j}^{+}\;\right)+a_{i,j}^{T}\left(\;-y_{i,j}-z_{i,j}\;\right)\;\right|\leq a_{i,j}^{T}x_{i,j}. \end{array}$$

This can be seen by considering Figure 5, from which we can deduce that region 3 has to lie between regions 1 and 2. By using the critical values of these adjacent regions, which can be determined from (60) and (62), we arrive at the inequality above.

For the reformulation of the condition with respect to *ρ*_2_^(1)^(**w**_0 *i*,*j*_^+^), we consider the different situations in which the boundary values of the corner regions can be for face number 6.

(i) If the boundary values are in situation S1 given in Table 2, the reformulation is given by
(66) $$\begin{array}{c} \rho_{2}^{\left(1\right)}\left(\;w_{0\;i,j}^{+}\;\right)+b_{i,j}^{T}\left(\;+x_{i,j}+y_{i,j}-z_{i,j}\;\right)\geq 0. \end{array}$$
(ii) If the boundary values are in one of the situations in the set *𝒮*_c_ defined in (63) or in situation S9.7 in Table 3, we get
(67) $$\begin{array}{c} \rho_{2}^{\left(1\right)}\left(\;w_{0\;i,j}^{+}\;\right)+b_{i,j}^{T}\left(\;-x_{i,j}-y_{i,j}-z_{i,j}\;\right)>0. \end{array}$$
(iii) If the boundary values are in one of the situations in *𝒮*_ac_ (64), the reformulation is
(68) $$\begin{array}{c} \rho_{2}^{\left(1\right)}\left(\;w_{0\;i,j}^{+}\;\right)+b_{i,j}^{T}\left(\;+x_{i,j}-y_{i,j}-z_{i,j}\;\right)>0. \end{array}$$

For the reformulation of the condition with respect to *ρ*_2_^(2)^(**w**_0 *i*,*j*_^+^), we consider this time the different situations belonging to face number 4.

(i) If the boundary values are in situation S1, the reformulation is given by
(69) $$\begin{array}{c} \rho_{2}^{\left(2\right)}\left(\;w_{0\;i,j}^{+}\;\right)+c_{i,j}^{T}\left(\;-x_{i,j}-y_{i,j}+z_{i,j}\;\right)\geq 0. \end{array}$$
(ii) If the situation of the boundary values is in the set *𝒮*_c_ or corresponds to the situation S9.7, we get
(70) $$\begin{array}{c} \rho_{2}^{\left(2\right)}\left(\;w_{0\;i,j}^{+}\;\right)+c_{i,j}^{T}\left(\;+x_{i,j}-y_{i,j}-z_{i,j}\;\right)>0. \end{array}$$
(iii) Finally, if the situation of the boundary values is in *𝒮*_ac_, the reformulation is
(71) $$\begin{array}{c} \rho_{2}^{\left(2\right)}\left(\;w_{0\;i,j}^{+}\;\right)+c_{i,j}^{T}\left(\;-x_{i,j}-y_{i,j}-z_{i,j}\;\right)>0. \end{array}$$

Finally, the reformulation of the conditions for cases 6, 9, 12, 13, 14, 16, 17, 22, 23, 25, and 26 in Table 1 is achieved similarly to this case.

*Case  15 and Similar Ones.* For this group of cases we will again make use of the different situations of the boundary values, but this time only for face number 6.

For the reformulation of the condition with respect to *ρ*_1_(**w**_0 *i*,*j*_^+^), we get the following list:

(i) If the boundary values are in situation S1
(72) $$\begin{array}{c} \left|\;\rho_{1}\left(\;w_{0\;i,j}^{+}\;\right)+a_{i,j}^{T}\left(\;-z_{i,j}\;\right)\;\right|<-a_{i,j}^{T}\left(\;+x_{i,j}+y_{i,j}\;\right). \end{array}$$
(ii) If the situation of the boundary values is in the set *𝒮*_c_
(73) $$\begin{array}{c} \left|\;\rho_{1}\left(\;w_{0\;i,j}^{+}\;\right)+a_{i,j}^{T}\left(\;-z_{i,j}\;\right)\;\right|\leq -a_{i,j}^{T}\left(\;-x_{i,j}+y_{i,j}\;\right). \end{array}$$
(iii) If the boundary values are in situation S9.7
(74) $$\begin{array}{c} \left|\;\rho_{1}\left(\;w_{0\;i,j}^{+}\;\right)+a_{i,j}^{T}\left(\;-z_{i,j}\;\right)\;\right|<-a_{i,j}^{T}\left(\;+x_{i,j}-y_{i,j}\;\right). \end{array}$$
(iv) If the situation of the boundary values is in the set *𝒮*_ac_
(75) $$\begin{array}{c} \left|\;\rho_{1}\left(\;w_{0\;i,j}^{+}\;\right)+a_{i,j}^{T}\left(\;-z_{i,j}\;\right)\;\right|\leq -a_{i,j}^{T}\left(\;-x_{i,j}-y_{i,j}\;\right). \end{array}$$

For the reformulation of the condition with respect to *ρ*_2_^(1)^(**w**_0 *i*,*j*_^+^), we get the following list:

(i) If the boundary values are in situation S1
(76) $$\begin{array}{c} \left|\;\rho_{2}^{\left(1\right)}\left(\;w_{0\;i,j}^{+}\;\right)+b_{i,j}^{T}\left(\;-z_{i,j}\;\right)\;\right|<-b_{i,j}^{T}\left(\;+x_{i,j}+y_{i,j}\;\right). \end{array}$$
(ii) If the situation of the boundary values is in the set *𝒮*_c_
(77) $$\begin{array}{c} \left|\;\rho_{2}^{\left(1\right)}\left(\;w_{0\;i,j}^{+}\;\right)+b_{i,j}^{T}\left(\;-z_{i,j}\;\right)\;\right|\leq -b_{i,j}^{T}\left(\;-x_{i,j}-y_{i,j}\;\right). \end{array}$$
(iii) If the boundary values are in situation S9.7
(78) $$\begin{array}{c} \left|\;\rho_{2}^{\left(1\right)}\left(\;w_{0\;i,j}^{+}\;\right)+b_{i,j}^{T}\left(\;-z_{i,j}\;\right)\;\right|<-b_{i,j}^{T}\left(\;-x_{i,j}+y_{i,j}\;\right). \end{array}$$
(iv) If the situation of the boundary values is in the set *𝒮*_ac_
(79) $$\begin{array}{c} \left|\;\rho_{2}^{\left(1\right)}\left(\;w_{0\;i,j}^{+}\;\right)+b_{i,j}^{T}\left(\;-z_{i,j}\;\right)\;\right|\leq -b_{i,j}^{T}\left(\;+x_{i,j}-y_{i,j}\;\right). \end{array}$$

Finally, we get for the reformulation of the condition with respect to *ρ*_2_^(2)^(**w**_0 *i*,*j*_^+^) the following list:

(i) If the boundary values are in situation S1
(80) ρ22w0 i,j++min⁡ci,jT−xi,j−yi,j−zi,j,ci,jT+xi,j+yi,j−zi,j>0.
(ii) If the situation of the boundary values is in the set *𝒮*_c_ or *𝒮*_ac_


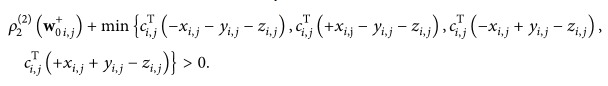

(81)

(iii) If the boundary values are in situation S9.7
(82) ρ22w0 i,j++min⁡ci,jT+xi,j−yi,j−zi,j,ci,jT−xi,j+yi,j−zi,j>0.

The reformulation of the conditions for cases 18, 19, 20, 24, and 27 in Table 1 is done then similarly to case 15 explained above.

*Case  21.* The last case which is left is case number 21. The corresponding region contains the part of the cube which was not covered so far with the corresponding regions of the other cases. Additionally, since it is unfortunately also possible that there are overlappings for some of the regions which appear at different faces, as, for example, the possible overlapping of region 1 with region 11, these overlappings are also assigned to case number 21 to finally achieve proper partitioning.

One could get the impression that this handling of the overlappings is mathematically rather grubby, but to legitimate this we recall that an optimal solution of problem (28) will result in rather small values for *ρ*_1_(**w**_*i*,*j*_^+^), *ρ*_2_^(1)^(**w**_*i*,*j*_^+^), and *ρ*_2_^(2)^(**w**_*i*,*j*_^+^) and therefore should be ideally close to case 21 in Table 1. Extending therefore the region for case number 21 should not be harmful. Furthermore, for small values of *ρ*_1_(**w**_*i*,*j*_^+^), *ρ*_2_^(1)^(**w**_*i*,*j*_^+^), and *ρ*_2_^(2)^(**w**_*i*,*j*_^+^) a reformulation of the condition can lead more likely to overlappings of certain region parts. Therefore it seems to be adequate to assign these overlappings to region 21.

It is possible to define region 21 in mathematical terms, but since we explained the content of this region already above this will be rather uninspiring and for the implementation an explicit formulation of the region is also not needed, since it can easily be depicted with the help of the other regions.

### 3.5. Resolvent Operators for Problem (12)

This section is very similar to Section 3.4 and we mainly have to just replace **w** ^+^ by **w** ^−^ and $\tilde{u}$ by $1-\tilde{u}$ . To achieve a smooth extension of **w** ^−^ to the domain Σ, we consider now instead of problem (12) the following problem:

(83) $$\begin{array}{c} \underset{w^{-}}{\min}\left\{\;\int_{\Omega }f\left(\;w^{-}\;\right)\left(\;1-\tilde{u}\left(\;x\;\right)\;\right)+\mu s\left(\;w^{-}\;\right)dx\;\right\}. \end{array}$$

The spaces *X* and *Y* and their inner products are defined in the same way as in Section 3.4 and the discretization of (83) is performed in the same manner as before.

The resolvent operator with respect to *F* ^*∗*^ is identical to (27). Finally, compared to the section before, there are only very slight changes of the resolvent operator with respect to *G*.

## 4. Implementation and Results

Although we have a convex minimization problem with respect to the motion segmentation function $\tilde{u}$ in (10) and linearised the fidelity term *f* in (8), we should keep in mind that the overall minimization problem remains nonconvex and that we have to update **w**_0_^+^ and **w**_0_^−^ regularly. Therefore a coarse-to-fine strategy is applied to avoid the risk of getting stuck in a local minimum during the optimisation. The minimal size of the images at the coarsest level is set to *n*_min_ = 32 and the scaling factor for the pyramid to *ξ* = 0.9. At the coarsest level we initialise the displacement fields **w** ^+^ and **w** ^−^ trivially with 0.

Because of Remark 1 in Section 2.2 one would expect that the choice of the initialisation for the function $\tilde{u}$ can be arbitrary. Indeed, whatever initialisation we choose, a global minimizer for the motion segmentation can be found for* fixed* **w** ^+^ and **w** ^−^. But in our case the values for **w** ^+^ and **w** ^−^ are updated regularly during optimisation.

The final iteration scheme consists of two loops. The outer loop iterates over the pyramid levels. In each level an inner loop updates the values for $\tilde{u}$ , **w** ^+^, and **w** ^−^, following steps (1)–(3) in Section 3.1. Thus, in each iteration of this inner loop one step of the primal-dual Algorithm 1 from [17] is performed with the appropriate resolvent operators from the previous sections to update $\tilde{u}$ , **w** ^+^, and **w** ^−^. To achieve a better convergence of $\tilde{u}$ we update **w** ^+^ and **w** ^−^ only every 50th iteration. The motion segmentation Σ is obtained by choosing *η* = 0.5 and setting $\Sigma =\Sigma (\eta )≔\left\{x\in \Omega |\tilde{u}\left(x\right)\geq \eta \right\}$ (see Remark 1 in Section 2.2).

As soon as the finest level is reached, the inner loop is executed until a certain tolerance or the maximum number of iterations is reached. The final displacement field is then obtained by setting

(84) $$\begin{array}{c} w\left(\;x\;\right)=\left\{\begin{array}{cc} w^{+}\left(x\right) & \text{if }x\in \Sigma ,\\ w^{-}\left(x\right) & \text{if }x\in \Omega \setminus \Sigma . \end{array}\right. \end{array}$$

Bicubic interpolation is used to calculate the images *T*(**x** + **w** ^±^) during the iterations and to obtain the final registered image *T*(**x** + **w**).

**Figure 10:**
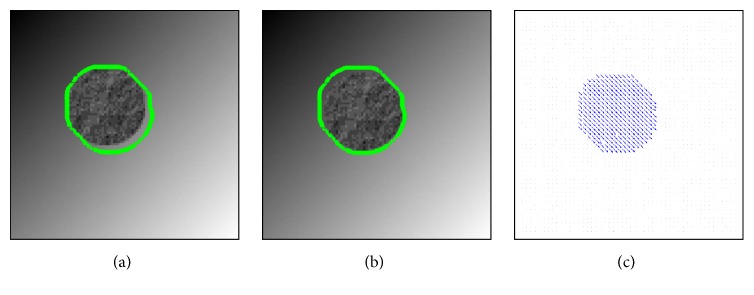
The template (a) and the reference (b) images superimposed with the motion segmentation (green curves) and the displacement field **w** (c). The parameter values used for this example: *γ*_1_ = 4, *γ*_2_ = 4, *μ* = 0.5, and *ν* = 0.3.

**Figure 11:**
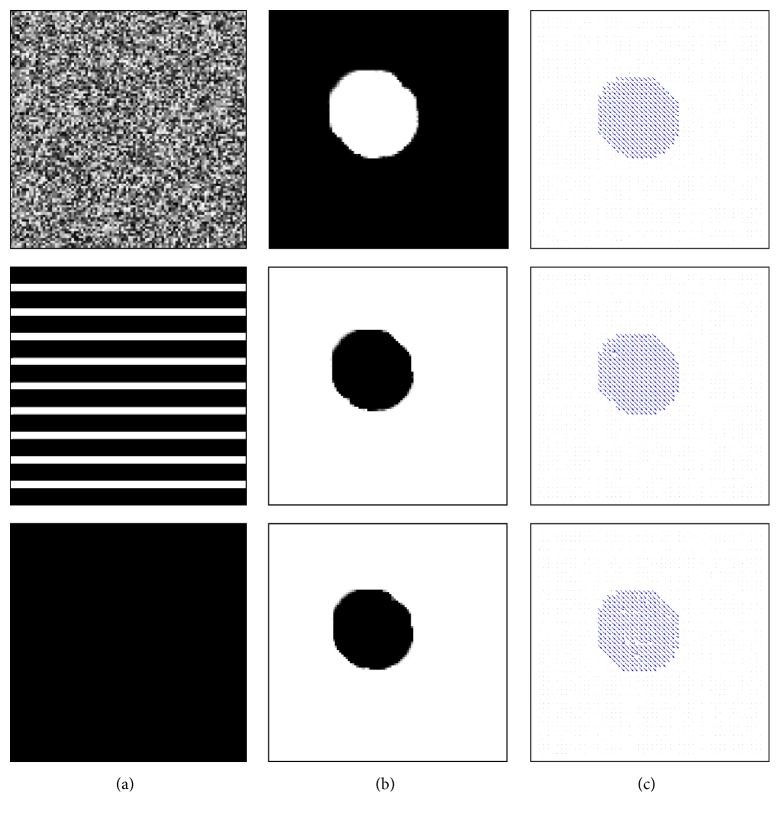
The motion segmentation results $\tilde{u}$ (b) and the corresponding displacement field results **w** (c) obtained when using different initialisations for $\tilde{u}$ (a) for the example and parameters in Figure 10. For the results in Figure 10 itself the reference image was used for initialisation.

**Figure 12:**
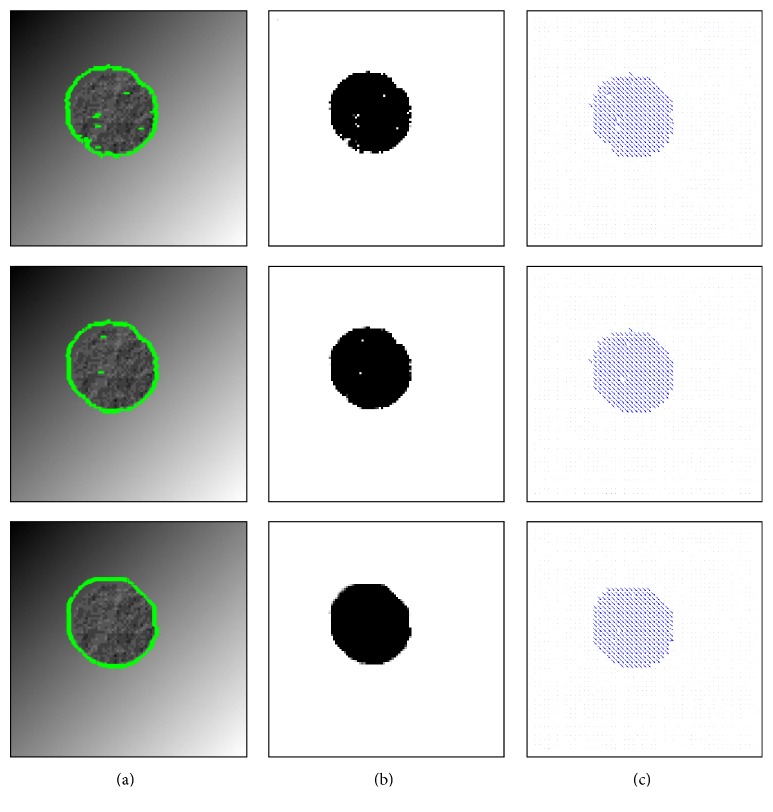
From top to bottom: the effect of the smoothness parameter *ν* when *ν* = 0.01, 0.05, and 0.2. Here we show the reference image superimposed with the contour of thresholded motion segmentation $\tilde{u}$ (a), $\tilde{u}$ (b), and the corresponding displacement field **w** (c) for the example in Figure 10. The other parameters are again *γ*_1_ = 4, *γ*_2_ = 4, and *μ* = 0.5.

In Figure 10, we show the registration result of a synthetic example, where a textured circle is moving down diagonally. Although our energy functional is convex with respect to the motion segmentation function $\tilde{u}$ , the overall minimization task remains a nonconvex problem. To decrease the influence of the nonconvexity during the minimization procedure, certain terms are linearised and a coarse-to-fine strategy is applied. Experiments for the synthetic example in Figure 10 show that due to this workaround different initialisation for $\tilde{u}$ could be used to achieve similar results, as shown in Figure 11. In order to show the effect of the smoothness parameter *ν* for the TV norm, we show in Figure 12 that we can obtain smoother results as *ν* increases.

In order to show the performance of the proposed method, we show qualitative and quantitative results. Since we are interested in the discontinuities of the displacement field, we compare the proposed method to methods that are able to preserve discontinuities in the displacement field, in this case, the demon algorithm with anisotropic diffusion filtering [26], the registration algorithm of Brox et al. [8], and our previous work [16].

**Figure 13:**
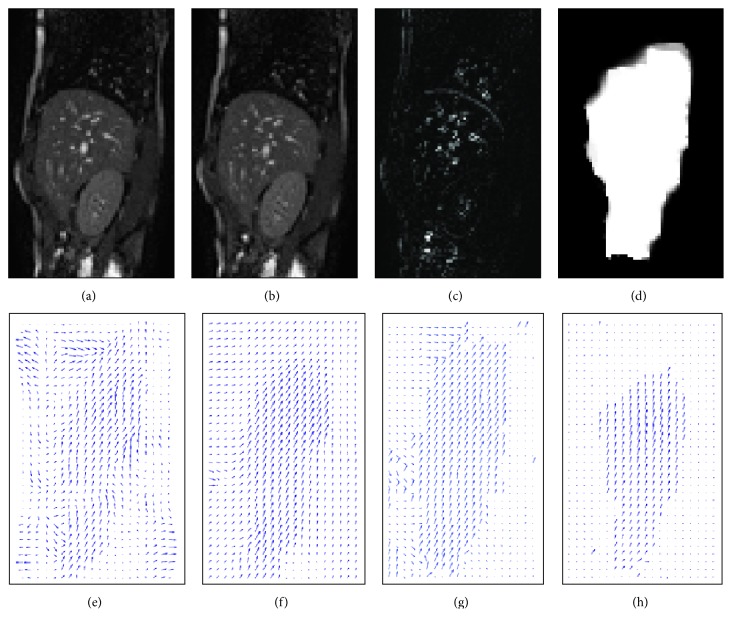
The template image (a), the reference image (b), the difference image (c), and the motion segmentation result (d). The displacement field for the demon algorithm with anisotropic diffusion filtering is shown in (e), the one for the registration algorithm of Brox et al. in (f), the one for our previous method in (g), and finally the one of our method in (h).

We show the qualitative comparison in Figure 13. As clearly seen, the proposed method can achieve more crisp discontinuities in the displacement field compared to the demon algorithm with anisotropic diffusion filtering and the registration algorithm of Brox et al. Furthermore, the proposed method managed nicely to separate the motion of the abdominal wall and the one of the organs.

**Figure 14:**
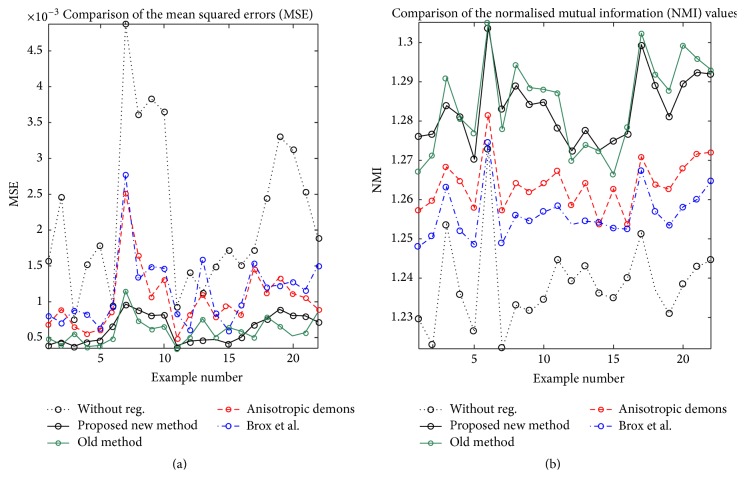
Quantitative evaluation for 22 pairs of liver images with a discontinuous displacement field. Comparison of the MSE (a) and NMI values (b).

In Figure 14 a quantitative evaluation is shown for 22 different liver image pairs from the sequences of [3]. We chose the parameters for all the methods by optimising them with respect to these image pairs. The parameters of our method were set to *γ*_1_ = 4, *γ*_2_ = 1, *μ* = 0.2, and *ν* = 0.1. For the demon algorithm with anisotropic diffusion filtering we could use the suggested parameters, for Brox et al.'s method we used *γ* = 5, *α* = 80, and *σ* = 0.9, and finally for our previous method we used the parameters suggested there, namely, *γ* = 0.4, *μ* = 0.05, *λ* = 0.05, *θ* = 4, and *ϵ* = 0.00001.

To quantitatively assess the performance of the four methods, we calculated the mean squared error (MSE) and the normalised mutual information (NMI), with the grey values scaled from 0 to 1. For all our examples the proposed method performed better than the demon algorithm with anisotropic diffusion and the registration algorithm of Brox et al. We used the Kolmogorov-Smirnov test to check for normality of the results using the R Software package (Version 2.10.1). We considered a significance level of 5% as significant. The *t*-test showed that the proposed method delivered significantly better results than the demon algorithm with anisotropic diffusion filtering and the method of Brox et al. with both *p* < 0.05. There was however no significant difference in the performance of the proposed method compared to our previous method [16] for the MSE and NMI.

In a next step we compared the running times of the proposed method to the ones of our previous work [16] for the 22 liver image pairs. Both methods were implemented in MATLAB and the experiments were performed on a 64-bit Linux system with 1.2 GHz. We used again the parameters *γ*_1_ = 4, *γ*_2_ = 1, *μ* = 0.2, and *ν* = 0.1 for the proposed method and for our previous work the parameters *γ* = 0.4, *μ* = 0.05, *λ* = 0.05, *θ* = 4, and *ϵ* = 0.00001. In [16] the displacement fields **w** ^+^ and **w** ^−^ were updated every 10th iteration. To provide a fair comparison, we therefore used the same update frequency for the proposed new method. Furthermore, the maximum number of iterations in the finest level is set to 30000 for both methods. The timing results for the 22 liver image pairs are shown in Table 4 together with the mean and standard deviation. The running times of both methods are comparable and the proposed new method performed with around 100 s slightly faster than the old method. The old method makes use of some optimised in-built MATLAB routines, whereas for the proposed method there is still a high potential to optimise the code.

## 5. Conclusion

In this paper we presented a primal-dual method for discontinuity preserving nonrigid registration that makes use of the continuous cuts framework. The so-gained motion segmentation influences the motion estimation positively by sharpening the discontinuities in the displacement field. The minimization of the energy functional was implemented in a coarse-to-fine strategy and exploits the rapidity of the primal-dual algorithm studied in [17]. The experimental results demonstrated desirable performance of the proposed method in comparison with those of the demon algorithm with anisotropic diffusion filtering [26] and the registration algorithm of Brox et al. [8].

Large displacements of deforming organs can cause misregistrations even when using a coarse-to-fine approach. Brox and Malik proposed in a very recent publication [27] to include point correspondences from descriptor matching into the variational optical flow formulation. In future work we plan on similarly including known corresponding landmarks into the functional as hard constraints as we have preliminarily done in [28]. However, we plan on using our* Tracking the Invisible* approach [29] for locating the matching point correspondences.

## Figures and Tables

**Table 1:** This table indicates the numbering of the 27 cases (see (29)).

Case number	ρ_1_(**w**_*i*,*j*_^+^)⋄0	ρ_2_^(1)^(**w**_*i*,*j*_^+^)⋄0	ρ_2_^(2)^(**w**_*i*,*j*_^+^)⋄0
1	>	>	>
2	<	>	>
3	=	>	>
4	>	<	>
5	<	<	>
6	=	<	>
7	>	>	<
8	<	>	<
9	=	>	<
10	>	<	<
11	<	<	<
12	=	<	<
13	>	=	>
14	<	=	>
15	=	=	>
16	>	=	<
17	<	=	<
18	=	=	<
19	>	=	=
20	<	=	=
21	=	=	=
22	>	>	=
23	<	>	=
24	=	>	=
25	>	<	=
26	<	<	=
27	=	<	=

**Table 2:** Different possible situations for the values *A*_*v*_, *A*_*h*_, *B*_*v*_, *B*_*h*_, *C*_*v*_, *C*_*h*_, *D*_*v*_, and *D*_*h*_.

Number	*A*_*v*_⋄*D*_*v*_	*B*_*v*_⋄*C*_*v*_	*A*_*h*_⋄*D*_*h*_	*B*_*h*_⋄*C*_*h*_
S1	>	>	>	<
S2	>	>	=	$\left(\begin{array}{c} <\\ = \end{array}\right)$
S3	>	>	<	$\left(\begin{array}{c} >\\ <\\ = \end{array}\right)$
S4	=	$\left(\begin{array}{c} >\\ = \end{array}\right)$	>	<
S5	=	$\left(\begin{array}{c} >\\ = \end{array}\right)$	=	$\left(\begin{array}{c} <\\ = \end{array}\right)$
S6	=	$\left(\begin{array}{c} >\\ = \end{array}\right)$	<	$\left(\begin{array}{c} >\\ <\\ = \end{array}\right)$
S7	<	$\left(\begin{array}{c} >\\ <\\ = \end{array}\right)$	>	<
S8	<	$\left(\begin{array}{c} >\\ <\\ = \end{array}\right)$	=	$\left(\begin{array}{c} <\\ = \end{array}\right)$
S9	<	$\left(\begin{array}{c} >\\ <\\ = \end{array}\right)$	<	$\left(\begin{array}{c} >\\ <\\ = \end{array}\right)$

**Table 3:** Splitting of situation S9 in Table 2 into its parts.

Number	*A*_*v*_⋄*D*_*v*_	*B*_*v*_⋄*C*_*v*_	*A*_*h*_⋄*D*_*h*_	*B*_*h*_⋄*C*_*h*_
S9.1	<	>	<	>
S9.2	<	>	<	=
S9.3	<	>	<	<
S9.4	<	=	<	>
S9.5	<	=	<	=
S9.6	<	=	<	<
S9.7	<	<	<	>
S9.8	<	<	<	=
S9.9	<	<	<	<

**Table 4:** Comparison of the running times of the proposed method to the ones of our previous work [16] for 22 liver image pairs. The times are given in seconds.

Ex. number	Previous method [16]	Proposed new method
1	1055.13	959.33
2	1053.55	951.41
3	1060.33	948.92
4	1059.57	996.78
5	1052.58	951.85
6	1064.84	955.43
7	1070.07	990.57
8	1060.53	944.11
9	1082.90	949.47
10	1079.99	949.23
11	1073.25	954.15
12	1048.56	961.26
13	1059.35	956.23
14	1061.09	949.74
15	1065.24	952.35
16	1072.26	949.80
17	1075.99	980.85
18	1068.88	951.10
19	1071.08	985.62
20	1064.42	949.60
21	1057.28	946.05
22	1060.19	979.33

Mean	1064.41	959.69
Std.	9.03	15.76
